# Elderly criminal patients in Razi psychiatric hospital of tunis

**DOI:** 10.1192/j.eurpsy.2021.1015

**Published:** 2021-08-13

**Authors:** H. Jemli, R. Jomli, H. Rym, I. Bouslama, A. Ben Cheikh Ahmed, F. Nacef

**Affiliations:** 1 Psychiatry Department A, Razi Hospital, manouba, Tunisia; 2 Psychiatry A Department, Razi Hospital, Manouba, Tunisia; 3 Avicenne, manouba psychiatry, manouba, Tunisia

**Keywords:** Elderly, forensic psychiatry, criminality

## Abstract

**Introduction:**

Criminality has become of increasing concern in the practice of psychiatry. However, violence among elderly psychiatric patients is an underestimated and understudied phenomenon.

**Objectives:**

The aim of the study is to identify differences in the socio-demographic, clinical and criminological profiles between elderly criminals under treatment for psychiatric disorders and those not known to have mental disorders prior to the criminal offense in Tunisia.

**Methods:**

We present a retrospective study on twenty male criminal mental patients, aged sixty years or older, who were hospitalized in the Forensic Psychiatry Department of Razi Hospital during 18 years, following a dismissal for insanity under Article 38 of the Penal Code and Article 29 of Law 92/83 on Mental Health.

**Results:**

Prevalence was higher among elderly criminals without a known psychiatric history (2.42% versus 1.98%). The average age was roughly the same, around 73 years old.Neurological and cardiovascular histories were the most common in both groups. The criminal act was indicative of dementia in 8 cases. Criminal history was more frequent in elderly patients with a personal psychiatric history (55.5% versus 18.2%). Patients whose act was revelatory of their mental disorder committed more violent crimes (63.7% versus 44.4%) using blunt objects (71.4% versus 0%).The victim most often belonged to the aggressor’s family, particularly the spouse (87.5%).

**Conclusions:**

Screening for criminal risk factors in the elderly, early diagnosis of mental disorders and a comprehensive therapeutic project are necessary to prevent the risk of violent behaviour.

